# Exosomal lncRNA SCIRT/miR-665 Transferring Promotes Lung Cancer Cell Metastasis through the Inhibition of HEYL

**DOI:** 10.1155/2021/9813773

**Published:** 2021-07-24

**Authors:** Zhengyang Wang, Minmin Lin, Lulu He, Hongyan Qi, Jing Shen, Kejing Ying

**Affiliations:** ^1^Department of Pulmonary and Critical Care Medicine, Regional Medical Center for National Institute of Respiratory Diseases, Sir Run Run Shaw Hospital, School of Medicine, Zhejiang University, Hangzhou 310016, China; ^2^Cancer Center, Zhejiang University, Hangzhou, Zhejiang 310058, China; ^3^Department of Pathology and Pathophysiology, and Department of Medical Oncology of the Second Affiliated Hospital, Zhejiang University School of Medicine, Hangzhou 310058, China; ^4^Department of Pathology and Pathophysiology, and Department of Radiation Oncology of the Second Affiliated Hospital, Zhejiang University School of Medicine, Hangzhou 310058, China

## Abstract

Lung cancer remains the leading cause of cancer-related death worldwide. Recently, extracellular vesicles such as exosomes have attracted considerable interest both as a source for theranostic biomarkers and an essential participant in lung cancer progression. However, how specific exosomal cargos, such as noncoding RNAs, are selectively packaged into exosomes and promote lung cancer progression remains unclear. In this study, we identified miR-665 as the most elevated exosomal miRNA from both non-small-cell lung cancer (NSCLC) and small-cell lung cancer (SCLC) patients. We further demonstrated that lncRNA SCIRT was also increased in cancer cell exosomes and may facilitate the exosomal loading of miR-665 with the help of hnRNPA1. As a consequence, exosomal miR-665 promoted lung cancer cell invasion and migration by targeting Notch downstream transcription factor HEYL. In addition, we found that miR-665 and SCIRT were significantly upregulated in tumor tissue and plasma of patients with lung cancer, and both of them showed increased expression in metastatic disease samples. Our findings suggest that the exosomal transferring of miR-665 and SCIRT is a functional and mechanism-driven pathway that contributes to cancer progression and, thus, may provide novel diagnostic and therapeutic targets for lung cancer.

## 1. Introduction

Lung cancer is the most commonly diagnosed cancer and the leading cause of cancer-related mortality among both men and women worldwide [1]. Although great progress has been made in the management of lung cancer, such as EGFR tyrosine kinase inhibitors and ALK inhibitors, the prognosis of the disease remains poor owing to the presence of distant metastasis in more than half of the patients at the time of diagnosis [2]. Recent advances have suggested that the tumor microenvironment (TME) plays a central role in lung tumor growth and metastasis, including inflammation, angiogenesis, immune modulation, and therapy responses [3]. However, the underlying mechanisms of the complex interplay between cancer cells and TME, especially cell-to-cell communications, are still not fully understood.

Exosomes are a subset of extracellular vesicles (EVs) of endosomal origin with a size range of ∼40 to 160 nm [4]. Accumulated evidence shows that cancer-derived exosomes can facilitate the reprogramming and intercellular communications in TME by transferring specific molecules [4, 5]. Among the molecular components carried in exosomes, microRNAs have attracted great attention due to their critical roles in cancer-related gene regulation and signal pathways. It has been reported that many exosomal miRNAs (exo-miRNAs) are associated with lung cancer metastasis [6–8]. For instance, Zhang et al. [9] demonstrated that exosomal miR-193a-3p, miR-210-3p, and miR-5100 could promote invasion of lung cancer cells via STAT3-induced epithelial-mesenchymal transformation (EMT). He et al. [10] reported that exosomal miR-499a-5p from highly metastatic cancer cells could enhance cell migration and EMT via the mTOR pathway. Exosomes with low levels of miR-34c-3p contribute to lung cancer cell metastasis by activating integrin signaling [2]. Moreover, several studies using high-throughput approaches also revealed that the exo-miRNA expression pattern of lung cancer patients has dramatically changed even in the early stage of the disease, indicating a close correlation between altered miRNAs and cancer progression [11, 12].

On the other hand, although the function of intracellular miRNAs is known to be controlled by many mechanisms, including long noncoding RNA- (lncRNA-) mediated competing endogenous RNA (ceRNA) networks, how certain miRNAs are selectively recognized and loaded into exosomes remain illdefined. Early studies speculate a cellular amount-dependent way for miRNAs to be passively packaged into exosomes, while recent surveys highlight the existence of active mechanisms for selective miRNA releasing, such as sphingomyelinase-2- (nSMase2-) dependent and EXOmotifs-dependent pathways [13, 14]. More active sorting mechanisms need to be discovered to improve our understanding of exo-miRNA's biological functions, especially in cancer development.

In the current study, we analyzed the exo-miRNA composition of lung-cancer-associated malignant pleural effusions (MPEs) as they always contain malignant cells and are tightly correlated with disease progression. MiR-665 was identified as the most upregulated exo-miRNA from both non-small-cell lung cancer (NSCLC) and small-cell lung cancer (SCLC) patients. Further studies revealed that miR-665 entered into exosomes in an lncRNA SCIRT-dependent way, thereby promoting the invasion and migration of lung cancer cells.

## 2. Materials and Methods

### 2.1. Patients and Clinical Samples

Pleural effusion (PE) samples from 10 patients (3 with tuberculosis, 4 with lung adenocarcinoma, and 3 with SCLC) were collected in tubes without anticoagulants from Sir Run Run Shaw Hospital. Plasma samples from 41 healthy persons (18 normal and 23 pneumonia) with no previous medical history of cancer and 67 lung cancer patients were collected in EDTA anticoagulation tubes. All patients enrolled in the study gave their consent, which was approved by the Ethical Committee of Sir Run Run Shaw Hospital. A lung cancer diagnosis was confirmed by fluid cytological or cancer histopathological analysis. The diagnosis of TB was based on a clinical evaluation of pleural effusions or a pleural biopsy. Samples were spun in tubes at 1000 × g for 10 min and then at 7000 × g for 30 min. Supernatants were stored at −80°C.

### 2.2. Cell Culture

All cell lines were purchased from the Cell Bank of the Chinese Academy of Sciences (Shanghai, China). Human bronchial epithelial cell BEAS-2B and HBE were cultured in DMEM (Invitrogen, CA, USA) and RPMI 1640 (Invitrogen). Non-small-cell lung cancer cells H1975 and H1650 and small-cell lung cancer cell H446 were cultured in RPMI 1640 with 10% fetal calf serum. For the exosome study, the conditioned medium was supplemented with 10% vesicle-free FBS.

### 2.3. Exosome Isolation and Identification

Exosomes were isolated with differential centrifugations. Briefly, samples were centrifuged at 500 × g for 15 minutes, 2000 × g for 20 min, and 10,000 × g for 40 min to remove cell debris and large vesicles. The supernatant was further ultracentrifuged at 100,000 × g for 90 minutes. All the steps were carried out at 4°C. The isolated exosomes were resuspended in PBS, stained with 2% uranyl acetate, and visualized by transmission electron microscopy (TEM) (Tecnai G2 Spirit, FEI, Czech Republic) operated at 80.0 kV. The size and number of exosomes were monitored using a Zeta View system (Particle Metrix, Germany).

### 2.4. Western Blotting

Proteins were extracted from exosomes or cells using RIPA lysis buffer, and immunoblotting was performed as previously described [15]. Anti-CD63, anti-TSG101, anti-calnexin, anti-hnRNPA1, and anti-GAPDH were from Santa Cruz Biotechnology (Santa Cruz, CA, USA), and anti-HEYL was from Abcam (Cambridge, UK).

### 2.5. RNA Sequencing

Total RNA was isolated from exosomes using Trizol (Invitrogen Life Technologies, CA, USA). Exosomal RNA was subjected to small RNA or lncRNA sequencing using the HiSeq × 10 platform (Illumina, California, USA) by OE Biotech (Shanghai, China). The known miRNAs were identified by aligning against the miRBase v.21 database. After that, the unannotated small RNAs were analyzed by miRDeep2 software to predict novel miRNAs. Altered miRNAs and lncRNAs were identified with the threshold of *P* value < 0.05 and fold change ≥ 2.

### 2.6. Transfection and RT-qPCR

The transfection of the miRNA inhibitor, mimic, and negative controls was conducted using Lipofectamine 3000 (Invitrogen) according to the manufacturer's instructions. The siRNAs targeting human SCIRT or HEYL (GenePharma, China) were transfected into cells using Lipofectamine™ RNAiMAX (Invitrogen) following the manufacturer's recommended protocol. A luciferase-specific siRNA was used as the negative control. RNA analysis was performed as previously described [16]. Primers and siRNA sequences are shown in Supplementary Table 1.

### 2.7. Methyl Thiazol Tetrazolium Assay (MTT)

Cells were seeded in 96-well plates. MTT was added after the transfection of miRNA mimics or inhibitors for 48 h. After incubating at 37°C for 3-4 h, an ELISA microplate reader was used to record the absorbance at 490 nm.

### 2.8. Invasion Assay

H1650, H1975, or H446 cells (1 × 10^5^ cells) were seeded in the upper chamber of the transwell apparatus (Millipore, USA), the bottom of which was coated with Matrigel (Corning, USA). Then, 10% FBS was added to the bottom chamber. After 48 h incubation, invaded cells were fixed and stained with crystal violet. Images were captured under a microscope at ×100 magnification.

### 2.9. Wound-Healing Assay

Cells were grown in 6-well plates overnight. A scratch was made on the cell monolayer using a micropipette tip, and dead cells were discarded by washing with phosphate-buffered saline (PBS). The rates of cell migration were calculated at 0 h and 48 h.

### 2.10. Dual-Luciferase Reporter Assay

HEYL wild-type and mutated 3′-UTR were subcloned into the pGL3-basic vector (Promega, Madison, WI, USA) and cotransfected with miR-665 mimics or miR-NC into cells with Lipofectamine 3000 (Invitrogen). Relative luciferase activity was detected using the Dual-Luciferase Reporter Assay System (Promega).

### 2.11. *In Vivo* Metastasis Assay

Wild-type TU strain zebrafish were maintained at 28°C with a 14/10 day/night cycle. Embryos at 48 h after fertilization (hpf) were collected for injection. Lung cancer cells were stained with DiI (Invitrogen) according to the manufacturer's instructions, and then, 100–150 cells per embryo were injected into the yolk sac of embryos using a borosilicate glass needle. Cell migration was photographed and analyzed after 72 h under a fluorescent microscope (Nikon SMZ18).

### 2.12. Statistical Analysis

Data were expressed as means ± standard deviation (SD) of at least three separate experiments. Statistical data analysis was performed using the two-tailed Student's *t*-test and one-way analysis of variance. The Wilcoxon rank sum test was used for nonparametric data. The survival curves were analyzed using the Kaplan–Meier method. *P* < 0.05 was considered statistically significant.

## 3. Results

### 3.1. MiR-665 Is Upregulated in MPE-Derived Exosomes from Both NSCLC and SCLC Patients

**Figure 1:**
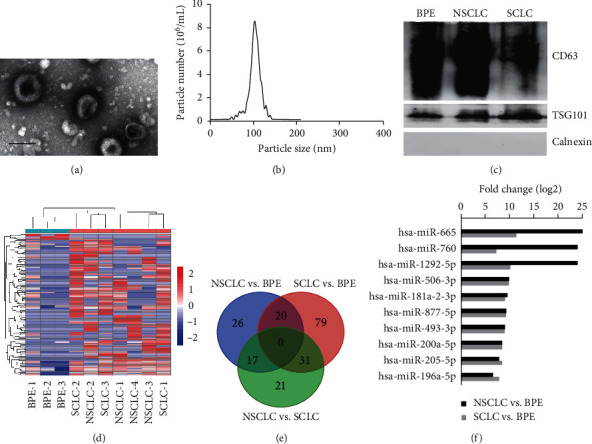
Identification of exosomal miRNAs associated with lung-cancer-induced MPE. (a) Electron microscopy analysis and (b) nanoparticle tracking analysis of exosomes from pleural effusions. Scale bar: 100 nm. (c) Western blotting analysis of CD63, TSG101, and calnexin in exosomes. (d) Cluster analysis heatmap of exosomal miRNAs from MPE (NSCLC and SCLC) and BPE samples. (e) The Venn diagram was used to identify overlapping and nonoverlapping exosomal miRNAs among NSCLC, SCLC, and TB effusions. (f) Upregulated exosomal miRNAs from both NSCLC and SCLC patients.

To explore exosomal communications in lung cancer progression, especially in metastasis, we isolated malignant pleural effusion- (MPE-) derived exosomes from NSCLC and SCLC patients. Benign pleural effusion- (BPE-) derived exosomes were also collected from tuberculous effusions, which is one of the principal differential diagnoses for malignant exudates in most developing countries. Exosomes were observed as round vesicles with intact membrane structures by electron microscopy (Figure 1(a)). Nanoparticle tracking analysis revealed that most of the particles in the isolated exosome samples had a size similar to those generally described for exosomes (i.e., 50–150 nm) (Figure 1(b)). Western blotting further confirmed the positive expression of exosome marker TSG101 and CD63 in our exosome preparations (Figure 1(c)). These results indicate that exosomes were successfully isolated from pleural effusions.

Then, we investigated the miRNA expression profiles of exosomes by high-throughput sequencing. Compared with the benign effusion group, 63 miRNAs (50 upregulated and 13 downregulated) and 130 miRNAs (96 upregulated and 34 downregulated) showed significantly altered levels in the exosomes of NSCLC and SCLC patients (2-fold change, *P* < 0.05), respectively (Figures 1(d) and 1(e)). Intriguingly, 20 differentially expressed miRNAs changed in common between NSCLC and SCLC (Figure 1(e) and Table s2), and 10 out of 16 upregulated miRNAs also exhibited increased expression in lung cancer tissues as confirmed by the dbDEMC 2.0 database (Figure s1(a)). Among them, miR-665 exhibited the most upregulated miRNA in both NSCLC and SCLC groups (Figure 1(f) and Table s2).

### 3.2. LncRNA SCIRT Facilitates the Loading of miR-665 into Exosomes

**Figure 2:**
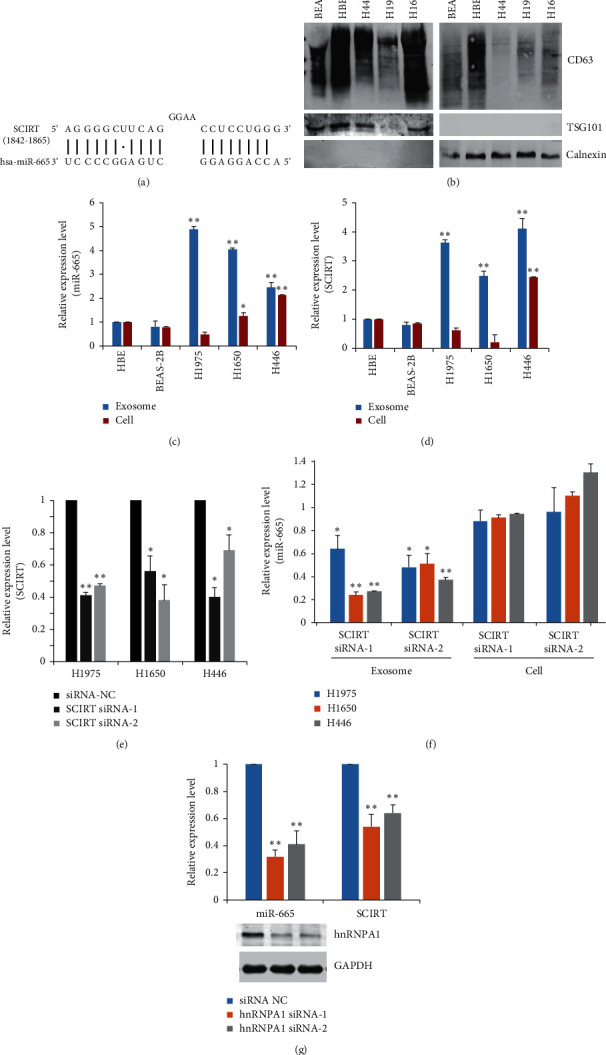
The exosomal loading of miR-665 relies on the facilitation of SCIRT and hnRNPA1. (a) Predicted binding sites between miR-665 and lncRNA SCIRT. (b) Western blotting analysis of CD63, TSG101, and calnexin in exosomes extracted from the cell culture supernatants and lung cancer cells. RT-qPCR analysis of the expression of (c) miR-665 and (d) SCIRT in cancer-cell-derived exosomes or lung cancer cells. (e) Knockdown of SCIRT with specific siRNAs in lung cancer cells. (f) RT-qPCR analysis of miR-665 expression after SCIRT silencing. (g) RT-qPCR analysis of the expression of miR-665 and SCIRT in H446 cells after hnRNPA1 silencing. The analyses were repeated three times, and the results were expressed as mean ± SD. ^*∗*^*P* < 0.05 and ^*∗∗*^*P* < 0.01.

LncRNAs play a key role in the functional regulation of miRNAs via interaction. To further explore the important lncRNAs involved in exosomal miRNA regulation, we identified lncRNAs with an increased level in MPE-derived exosomes and compared them with miR-665-interacting lncRNAs predicted by using miRanda and DIANA-LncBase software. A newly discovered lncRNA, SCIRT, was successfully identified (Figure 2(a)).

Next, we examined the expression of miR-665 and SCIRT in NSCLC (H1975 and H1650) and SCLC (H446) cells. Exosomes were extracted from the cell culture supernatants. Meanwhile, intracellular RNAs were also prepared (Figure 2(b)). Compared with the human bronchial epithelial cells (HBE and BEAS-2B), miR-665 and SCIRT showed elevated levels in cancer-cell-derived exosomes (Figures 2(c) and 2(d)). However, their intracellular expression was not always consistent with the exosomal expressions, indicating a specific enrichment of those cargos in cancer-cell-associated vesicles.

Then, we knocked down SCIRT with specific siRNAs in lung cancer cells. The exosomal level of miR-665 decreased significantly, while its cellular level kept nearly unchanged, supporting that lncRNA SCIRT regulates the exosomal loading of miR-665 (Figures 2(e) and 2(f)). To further investigate related sorting mechanisms, we analyzed the interaction between SCIRT or miR-665 sequence and motifs of RNA-binding proteins (RBPs) by using the RBPDB database. The results revealed that only SCIRT had specific binding sites for heterogeneous nuclear ribonucleoprotein A1 (hnRNPA1), which has been reported in controlling the sorting of miRNAs into exosomes. Interestingly, when we depleted the expression of hnRNPA1 by two independent siRNAs, the exosomal level of SCIRT and miR-665 both decreased accordingly (Figure 2(g)). These data indicate that SCIRT may facilitate the loading of miR-665 into cancer-cell-derived vesicles with the help of hnRNPA1.

### 3.3. Exosomal miR-665 Mainly Affects Lung Cancer Cell Invasion and Migration Rather Than Proliferation

**Figure 3:**
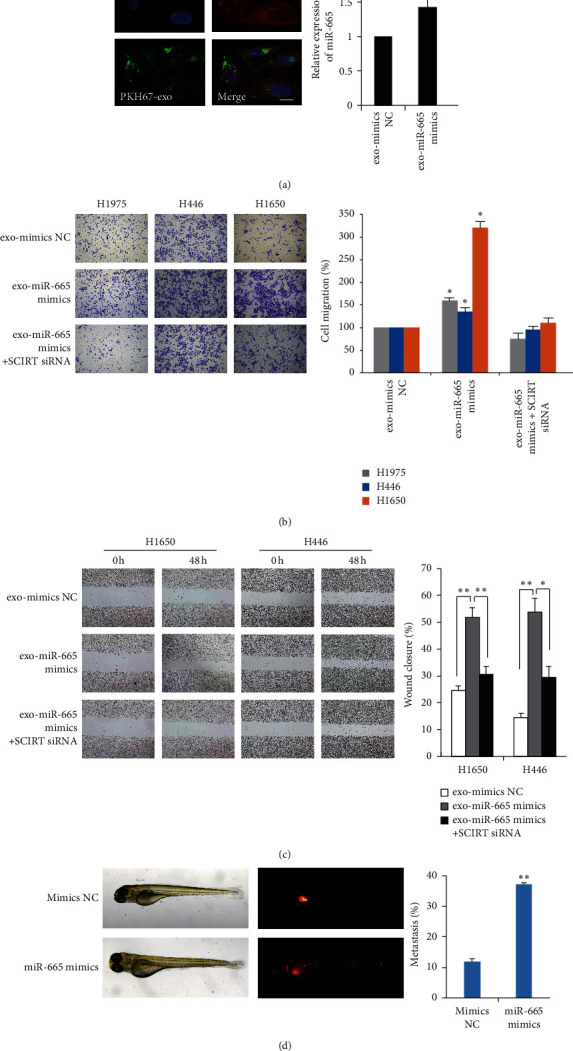
Exosomal miR-665 enhanced lung cancer cell invasion and migration. (a) Left: confocal microscopy of H446 cells (labeled with DiI, red) treated with H446 exosomes (labeled with PKH67, green). The nucleus of cells was labeled with DAPI (blue). Scale bar represents 10 *μ*m. Right: RT-qPCR analysis of miR-665 expression after exposing to exosomes from NC or miR-665 mimic transfected H446 cells. (b) Transwell invasion assay and (c) wound-healing assay of cells were performed following treatment with exosomes derived from cancer cells. ^*∗*^*P* < 0.05 and ^*∗∗*^*P* < 0.01, compared with the corresponding negative control (NC) group. (d) Fluorescence imaging of H446 cell (labeled with DiI, red) migration in zebrafish.

We further analyzed the biological function of exosomal miR-665 in lung cancer cells. Exosomes labeled with PKH67 were used to treat corresponding cells, and the cellular uptake of exosomes was observed and confirmed by fluorescence microscopy. MiR-665 was highly expressed in lung cancer cells after coculture with exosomes from miR-665 mimic transfected cells (exo-miR-665 mimic), indicating the efficient transferring of the miRNA (Figure 3(a)). Then, we monitored changes in cell proliferation, invasion, and migration after exosome incubation. The results showed that administration of exo-miR-665 mimics significantly promoted cell invasion and migration of both NSCLC and SCLC cells (Figures 3(b) and 3(c)), while it has no apparent effects on cell proliferation (Figure s1(b)). Moreover, cell invasion and migration ability were decreased when the expression of SCIRT was depleted (Figures 3(b) and 3(c)). Then, we investigated whether the exosomal miR-665 could contribute to the invasive capability of lung cancer cells *in vivo*. DiI-labeled H446 cells were injected into the zebrafish yolk sac, and the migration of the cancer cells to the tail was monitored. As a result, the number of fish showing metastasis is significantly greater in the miR-665 overexpression group compared with the control group (Figure 3(d)). Thus, these data suggested that exosomal miR-665 promoted lung cancer cell migration and invasion *in vitro* and *in vivo*.

### 3.4. HEYL Is a Novel Target of miR-665 in Lung Cancer Cells

**Figure 4:**
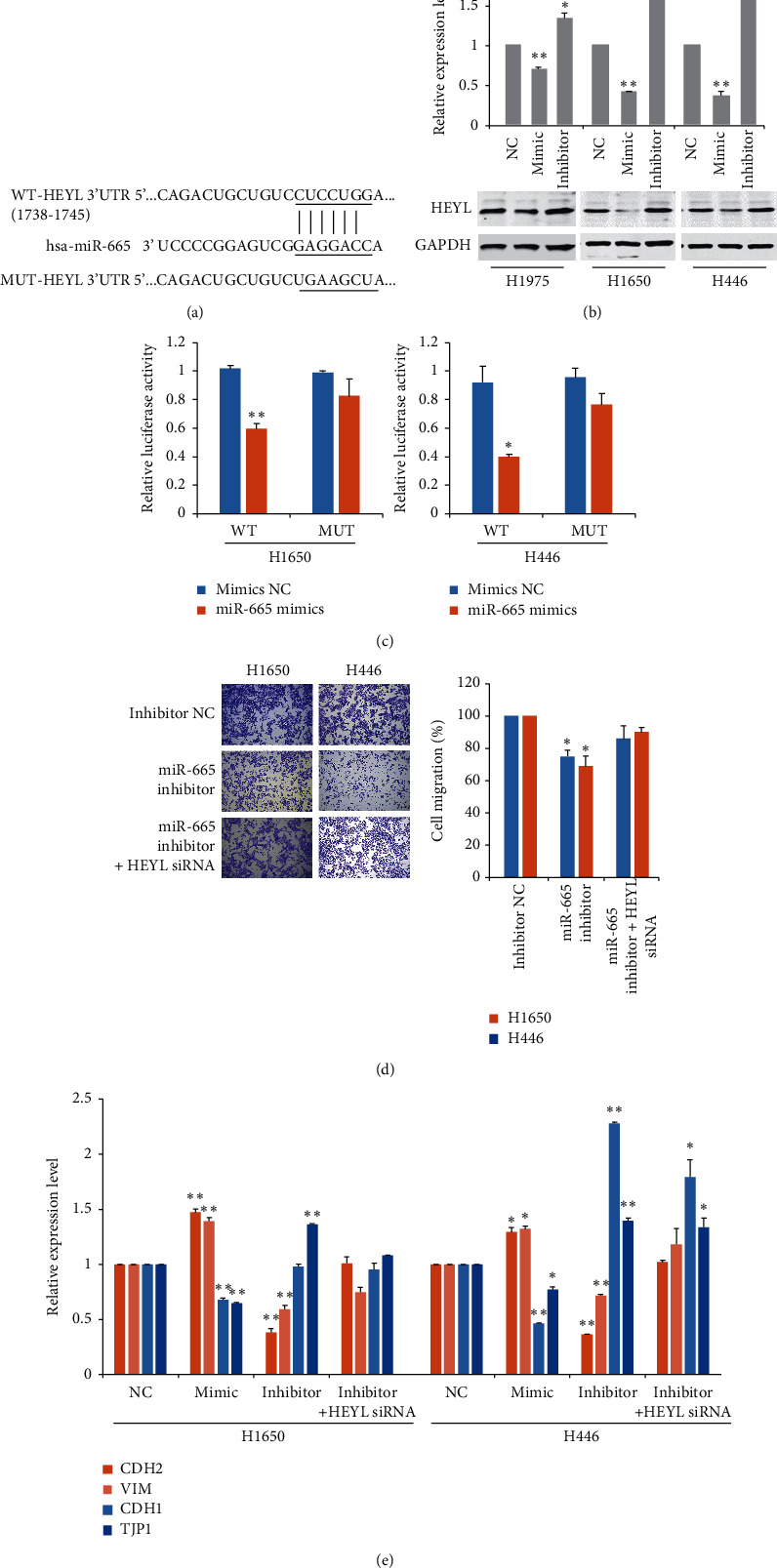
HEYL is the downstream target of miR-665 in lung cancer cells. (a) Schematic representation of the putative binding site of miR-665 in 3′-UTR of HEYL. (b) RT-PCR analysis of the expression of HEYL after administration of mimics or inhibitors of miR-665. (c) Dual-luciferase reporter assay was used to determine the effects of miR-665 expression on the activities of wild-type (WT) or mutant-type (MUT) HEYL 3′-UTR. (d) Transwell invasion assay of cells with or without HEYL knockdown was performed following treatment with exosomes derived from cancer cells. (e) RT-qPCR analysis of epithelial and mesenchymal markers after administration of mimics or inhibitors of miR-665. ^*∗*^*P* < 0.05 and ^*∗∗*^*P* < 0.01, compared with the corresponding negative control (NC) group.

Using TargetScan and miRanda software, we predicted miR-665-regulated molecular targets. A potential binding site was revealed for miR-665 in the 3′-UTR of HEY-like protein (HEYL) mRNA after sequence alignment, suggesting that miR-665 might regulate the expression of HEYL, a target gene of the Notch pathway (Figure 4(a)). Transfection of miR-665 mimics remarkably reduced the level of HEYL, whereas the administration of inhibitors significantly induced its expression (Figure 4(b)). Luciferase reporter assay was further performed to confirm a direct interaction between miR-665 and HEYL. Results showed that the luciferase activity was markedly decreased after ectopic miR-665 expression in cells with WT 3′-UTR of HEYL but not with the MUT 3′-UTR of HEYL (Figure 4(c)). Transwell assays also showed that the inhibitory effects of invasion caused by miR-665 inhibitors were reversed by HEYL silencing (Figure 4(d)).

It is well known that EMT plays a key role in cancer metastasis. Therefore, we analyzed the regulation effects of miR-665 on EMT-related markers. As shown in the results, transfection of miR-665 significantly decreased the level of epithelial markers such as E-cadherin and tight junction protein 1 (TJP1) and increased the expression of mesenchymal markers, including N-cadherin and vimentin (Figure 4(e)). In contrast, inhibitors of miR-665 showed inverse effects. In addition, HEYL suppression allows restoration of EMT-related genes, especially epithelial markers (Figure 4(e)). These results supported the findings that HEYL was a direct target of miR-665 and contributed to miR-665-mediated cancer cell invasion.

### 3.5. LncRNA SCIRT/miR-665/HEYL Pathway Is Dysregulated in Lung Cancer Patients

**Figure 5:**
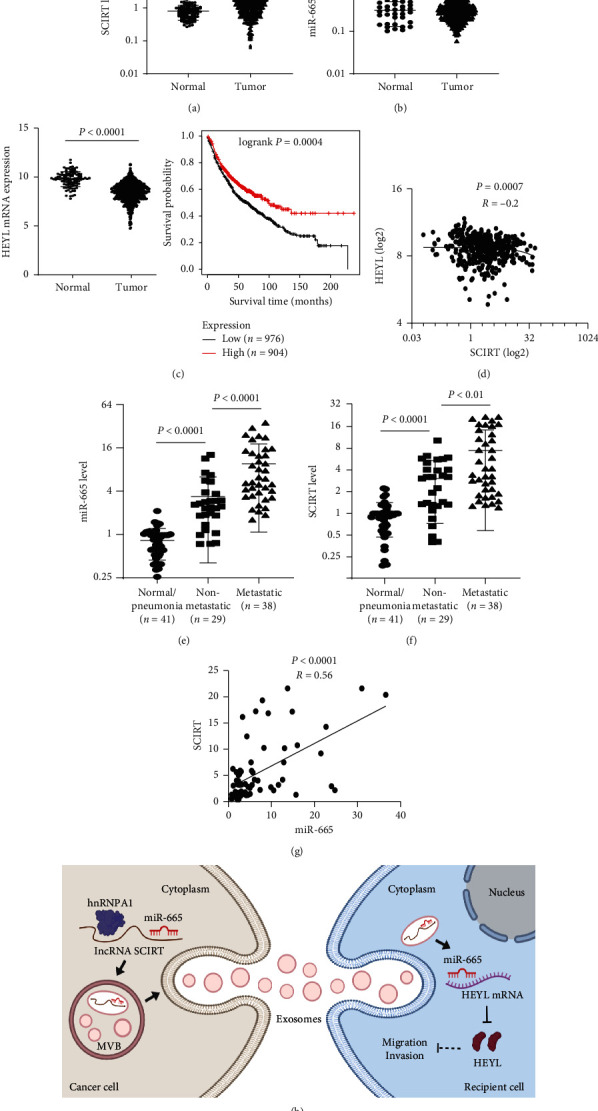
LncRNA SCIRT/miR-665/HEYL pathway is dysregulated in lung cancer patients. TCGA database analysis of the expression of (a) SCIRT, (b) miR-665, and (c) HEYL in lung cancer tissues. Kaplan–Meier survival curves by log-rank tests on lung cancer patients were stratified by HEYL expression levels for overall survival from the Kaplan–Meier plotter database. (d) The correlation between SCIRT and HEYL expression of the TCGA database. The plasma level of (e) exosomal miR-665 and (f) SCIRT in healthy persons and lung cancer patients. (g) The correlation between SCIRT and miR-665 expression of plasma-derived exosomes. (h) Schematic map of the lncRNA SCIRT/miR-665/HEYL pathway in lung cancer.

To determine the clinical relevance of the lncRNA SCIRT/miR-665/HEYL pathway, we analyzed The Cancer Genome Atlas (TCGA) database and identified significant upregulation of SCIRT and miR-665 and downregulation of HEYL in lung cancer tissues (Figures 5(a) and 5(c)). Among them, a negative correlation between the expression of SCIRT and HEYL was also identified (Figure 5(d)). Survival analysis showed that high expression levels of HEYL were positively correlated with the overall survival (OS) of the patients (Figure 5(c)).

Since exosomal noncoding RNAs have great cancer diagnostic and prognostic potentials, we further assessed the plasma level of miR-665 and SCIRT in healthy persons and lung cancer patients. We found that miR-665 and SCIRT were significantly upregulated in lung cancer plasma exosomes, and both of them showed increased expression in metastatic patient samples (Figures 5(e) and 5(f)). Moreover, there was a positive correlation between the plasma expression of miR-665 and SCIRT (Figure 5(g)).

## 4. Discussion

Accumulated evidence shows that cancer-derived exosomes play a versatile role in the formation and reprogramming of the tumor microenvironment [4, 5]. Meanwhile, although the aberrant expression of exosomal miRNAs is considered to be tightly involved in the pathogenesis of cancer, including metastasis, how specific miRNA species modulating intercellular communications in TME is still largely unknown. In the current study, we identified miR-665 as the most highly expressed miRNAs in MPE-derived exosomes from both NSCLC and SCLC patients. Further studies revealed that the exosomal loading of miR-665 relies on the facilitation of lncRNA SCIRT and RNA-binding protein hnRNPA1. Exosomes enriched with miR-665 subsequently entered recipient cells to promote cell invasion and migration by targeting Notch downstream transcription factor HEYL (Figure 5(h)).

MiR-665 has been reported to show diverse roles in cancer. For instance, miR-665 is downregulated in osteosarcoma [17], pancreatic cancer [18], and cervical cancer [19] and upregulated in hepatocellular carcinoma [20], breast cancer [21], and gastric adenocarcinoma [22]. In lung cancer, a recent study reported that the expression of miR-665 is elevated in NSCLC tissue and associated with a poor prognosis [23]. However, the role of exosomal-secreting miR-665 in lung cancer development remains unclear. In the current study, we revealed that miR-665 was enriched in extracellular vesicles in both NSCLC- and SCLC-related MPE and cell culture supernatants, thus promoting cancer cell invasion as well as migration. Meanwhile, we also demonstrated a significant increase of exosomal miR-665 levels in the plasma of lung cancer patients, especially in metastatic disease, suggesting a potential diagnostic role for this miRNA in cancer progression.

The recently discovered ceRNA regulatory networks between various types of noncoding RNA have gained considerable attention. Notably, miR-665 was found under the control of multiple ceRNA regulations. LncRNA DANCR and linc00462 activate the TGF/Smad signaling by suppressing miR-665 in cervical and pancreatic cancer [18, 19]. LncRNA BCAR4 maintains colorectal cancer cell stemness by targeting the miR-665/STAT3 pathway [24]. LncRNA MEG3 negatively regulates the expression of miR-665 in gastric cancer, which is followed by activation of the FAK/Src pathway [22]. Intriguingly, in this study, we uncovered another layer of lncRNA-mediated modulation of miR-665. A newly identified lncRNA, stem cell inhibitory RNA transcript (SCIRT), acts as the facilitator for exosomal loading of miR-665. SCIRT was reported to play a crucial role in cancer cell state transitions by direct interaction with chromatin modifier EZH2, then inhibiting cancer cells' self-renewal process [25]. Our findings that SCIRT might promote the selective sorting of critical exo-miRNAs into cancer cell exosomes further extend our understanding of its role in tumorigenesis. Besides, although we observed no significant changes in cancer cell proliferation, invasion, and migration after overexpression or silencing SCIRT, whether SCIRT could affect other steps of lung cancer metastasis requires further elucidation. Moreover, we also revealed a positive regulation of RNA-binding protein hnRNPA1 in exosomal SCIRT/miR-665 secretion. In exosomes, several RBPs have been shown to be involved in exo-miRNA export by binding specific motifs, such as hnRNPA2B1, hnRNPA1, and hnRNPQ [26–28]. Our results raise the possibility that RBPs may also mediate exo-miRNA releasing by targeting miRNA-interacted lncRNAs. Further studies are warranted to illustrate the exact underlying mechanisms and how SCIRT and miR-665 are selectively packaged into cancer exosomes.

Cancer cells undergoing EMT are associated with metastatic properties by enhancing cell mobility, invasion, and resistance to apoptotic stimuli [29]. Previous studies focusing on the oncogenic role of miR-665 in cancer indicated a promotion impact of miR-665 on the EMT process [20–22]. However, the potential molecular mechanisms remain elusive. By several lines of evidence, we confirmed that HEYL is a novel target of miR-665 in lung cancer cells. Silencing HEYL efficiently reversed the inhibitory effects of cell migration and invasion caused by miR-665 inhibitors and showed a direct influence on EMT-related genes, especially epithelial markers. It is known that HEYL proteins (HEY1, HEY2, and HEYL) belong to the hairy and enhancer of split-related (HESR) family and are downstream targets of the Notch pathway [30]. Since Notch signaling is involved in the metastatic spread of various tumors, HEYL is also supposed to participate in the progression and metastasis of cancer. Some studies identified HEYL as a potential tumor suppressor, such as promoting P53-mediated apoptosis in hepatocellular carcinoma and inhibiting prostate cancer cell growth [31]. A recent report also indicated that a single-nucleotide polymorphism (SNP) in the promoter of HEYL was significantly associated with the worse survival of NSCLC patients [32]. For the molecular function, HEY proteins typically act as repressive transcription factors by forming homo- or heterodimers to regulate gene expression [33]. Intriguingly, by using bioinformatic analysis, we also identified several potential HEY-binding sites in the promoter of EMT marker genes, suggesting a direct transcriptional regulatory mechanism might exist.

In summary, the present study highlights the role of exosomal miR-665 in promoting lung cancer cell invasion and metastasis by directly repressing Notch target gene HEYL. Importantly, our data also revealed that lncRNA SCIRT and the hnRNPA1 protein might mediate miR-665 packaging into cancer-cell-derived exosomes. In addition, we identified that plasma exosomal miR-665 and SCIRT are significantly upregulated in lung cancer patients and correlated with the advanced stage of the disease, therefore indicating a diagnostic and prognostic potential of the regulatory pathway.

## Data Availability

All data used during the present study are available from the corresponding author upon reasonable request.

## Supplementary Materials

Supplementary Materials. Figure S1. (a) The expression of miRNAs in the dbDEMC 2.0 database and (b) cell proliferation analysis after treating cells with exosomes derived from cancer cells. Table S1. A list of primers used in this study. Table S2. Differentially expressed exosomal miRNAs.
