# Supplementary material for: Exosomal lncRNA SCIRT/miR-665 Transferring Promotes Lung Cancer Cell Metastasis through the Inhibition of HEYL
Source: J Oncol. 2021 Jul 24;2021:9813773. doi: 10.1155/2021/9813773 (PMC8328715; doi:10.1155/2021/9813773)
Supplement: Supplementary Materials. — Figure S1. (a) The expression of miRNAs in the dbDEMC 2.0 database and (b) cell proliferation analysis after treating cells with exosomes derived from cancer cells. Table S1. A list of primers used in this study. Table S2. Differentially expressed exosomal miRNAs. [file 9813773.f1.docx]

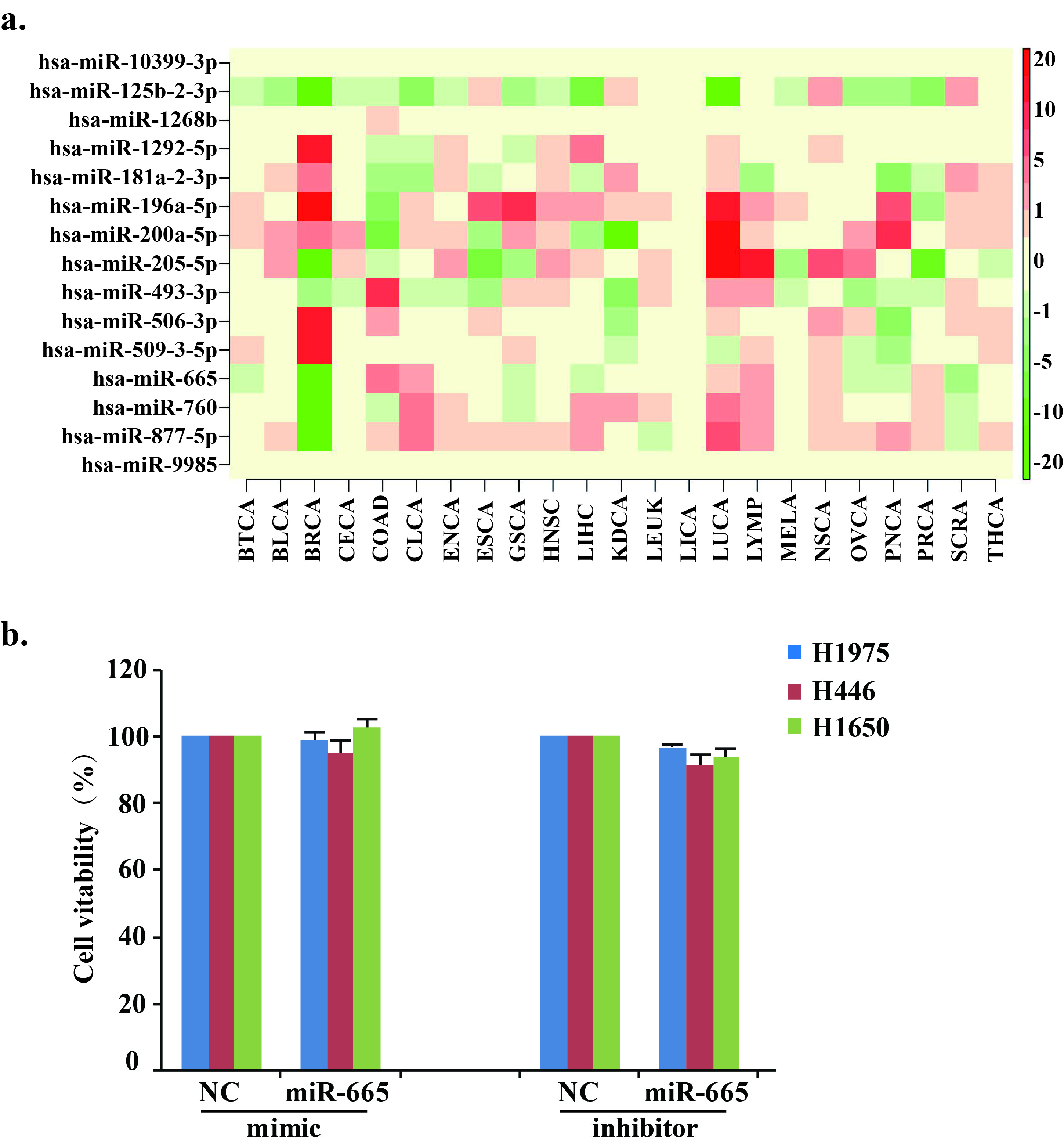


Figure S1. (a) The expression of miRNAs in human cancer tissues in dbDEMC 2.0 database. BTCA, Biliary tract cancer; BLCA, Bladder cancer; BRCA, Breast cancer; CECA, Cervical cancer; COAD, Colon cancer; CLCA, Colorectal cancer; ENCA, Endometrial cancer; ESCA, Esophageal cancer; GSCA, Gastric cancer; HNSC, Head and neck cancer; LIHC, Hepatocellular carcinoma; KDCA, Kidney cancer; LEUK, Leukemia; LICA, Liver cancer; LUCA, Lung cancer; LYMP, Lymphoma; MELA, Mesothelioma; NSCA, Nasopharyngeal cancer; OVCA, Ovarian cancer; PNCA, Pancreatic cancer; PRCA, Prostate cancer; SCRA, Sarcoma; THCA, Thyroid cancer. (b) Cell proliferation was determined by MTT assay after treating cells with exosomes derived from cancer cells.

Supplementary Table 1. A list of primers used in this study.

| Primers | Sequence |
| --- | --- |
| miR-665 | 5’-ACCAGGAGGCTGAGGCCCCT-3’ |
| CDH2 forward | 5’-TGACTCCAACGGGGACTGCACA-3’ |
| CDH2 reverse | 5’-CTGGCGTTCTTTATCCCGGCGT-3’, |
| VIM forward | 5’-CCAAGAACCTGCAGGAGGCAGAAG-3’ |
| VIM reverse | 5’-GCATCTGGCGTTCCAGGGACTCAT-3’ |
| CDH1 forward | 5’-AAGCCCCCATCTTTGTGCCTCCT-3’ |
| CDH1 reverse | 5’-TCCAGCCAGTTGGCAGTGTCTCT-3’ |
| TJP1 forward | 5’-GCACAGTTTGGCACAGCCTCCT-3’ |
| TJP1 reverse | 5’-AGCACGCCCCCATTGCTGTT-3’ |
| HEYL forward | 5’-CAAGCATGCAACTCCAAAGA-3’ |
| HEYL reverse | 5’-AGGAAGGCTTGGGGATAGAA-3’ |
| SCIRT forward | 5’- CTTGCCACTTCTTCTGGATG -3’ |
| SCIRT reverse | 5’- TCCTAGCTACTTATCCTAGCCT -3’ |
| Control siRNA | 5’-CUUACGCUGAGUACUUCGATT-3’ |
| SCIRT siRNA-1 | 5’-GUUUGUAGAUGUAAUCAAA-3’ |
| SCIRT siRNA-2 | 5’-CACUGUUGUUGGUUGAAUU-3’ |

Supplementary Table 2. Differentially expressed exosomal miRNAs in patients with lung cancer-associated MPE compared to individuals with BPE.

| **miRNA** | **log_2_(NSCLC/BPE)** | **log_2_(SCLC/BPE)** | **Sequence** |
| --- | --- | --- | --- |
| hsa-miR-665 | 25.07 | 11.37 | ACCAGGAGGCTGAGGCCCCT |
| hsa-miR-1292-5p | 24.08 | 10.14 | TGGGAACGGGTTCCGGCAGACGCTG |
| hsa-miR-760 | 24.08 | 7.23 | CGGCTCTGGGTCTGTGGGGA |
| hsa-miR-506-3p | 9.87 | 9.83 | TAAGGCACCCTTCTGAGTAGA |
| novel278 | 9.85 | 9.20 | GAAAAGGGAGGGAAAAGGAA |
| hsa-miR-181a-2-3p | 9.53 | 9.03 | ACCACTGACCGTTGACTGTACC |
| hsa-miR-877-5p | 9.30 | 9.26 | GTAGAGGAGATGGCGCAGGG |
| novel34 | 9.09 | 8.87 | GGCTGCTGATCTGGAAGAGG |
| hsa-miR-493-3p | 9.01 | 8.97 | TGAAGGTCTACTGTGTGCCAGG |
| hsa-miR-200a-5p | 8.44 | 8.44 | CATCTTACCGGACAGTGCTGGA |
| hsa-miR-125b-2-3p | 8.26 | 9.83 | TCACAAGTCAGGCTCTTGGGAC |
| hsa-miR-1268b | 8.18 | 8.38 | CGGGCGTGGTGGTGGGGGTG |
| hsa-miR-205-5p | 7.83 | 8.43 | TCCTTCATTCCACCGGAGTCTG |
| novel105 | 7.17 | 7.70 | CGCCGTGGGGGGCGGGGCGG |
| hsa-miR-509-3-5p | 6.81 | 24.30 | TACTGCAGACGTGGCAATCATG |
| hsa-miR-196a-5p | 6.57 | 7.82 | TAGGTAGTTTCATGTTGTTGGG |
| novel227 | -6.63 | -6.91 | CAGCTGGGAGAGCACCTGC |
| novel59 | -7.27 | -8.96 | CTCTAGAGAACCATCTGAAAGA |
| hsa-miR-10399-3p | -8.08 | -8.16 | CTCTCGGACAAGCTGTAGGTC |
| hsa-miR-9985 | -8.26 | -8.56 | TTCACAGTGGCTAAGCTAT |
